# Foveal microvasculature features of surgically closed macular hole using optical coherence tomography angiography

**DOI:** 10.1186/s12886-017-0607-z

**Published:** 2017-11-28

**Authors:** Joon Hee Cho, Ho Chul Yi, So Hyun Bae, Hakyoung Kim

**Affiliations:** Department of Ophthalmology, Kangnam Sacred Heart Hospital, Hallym University School of Medicine, 1, Singil-ro, Yeongdeungpo-gu, Seoul, 150-950 South Korea

**Keywords:** Idiopathic macular hole, Optical coherence tomography angiography, Superficial capillary plexus, Deep capillary plexus, Vitrectomy

## Abstract

**Background:**

To describe the features of foveal microvasculature using optical coherence tomography angiography (OCTA) and to determine the related clinical factors in eyes with surgically closed macular hole (MH).

**Methods:**

A retrospective case series of 18 patients with unilateral MH was reviewed. The patients maintained complete hole closure after vitrectomy with inner limiting membrane (ILM) peeling for at least 12 months. The healthy fellow eyes were studied as controls. The foveal microvasculature of both eyes was examined by OCTA. The area of the foveal avascular zone (FAZ) and the vascular density (VD) ratio in the superficial and deep capillary plexuses (SCP and DCP) were determined after surgery. Several clinical factors including age, stage and dimensions of MH, papillofoveal distance, the extent of nasal displacement of the fovea after surgery, postoperative central foveal thickness, and outer-retina integrity were evaluated to determine any relationships with the OCTA parameters.

**Results:**

The mean FAZ area in both the SCP and DCP (0.29 ± 0.11 mm^2^ and 0.39 ± 0.14 mm^2^) was significantly smaller than those of the controls (0.45 ± 0.14 mm^2^ and 0.62 ± 0.22 mm^2^) (*p* = 0.001 and <0.001, respectively). The mean VD ratio in the SCP (0.270 ± 0.349) was similar to that of the controls (0.321 ± 0.189) (*p* = 0.231); however, that in the DCP (0.321 ± 0.189) was significantly lower than that of the controls (0.331 ± 0.119) (*p* = 0.025). Only the extent of nasal displacement of the fovea was correlated with the DCP FAZ-area difference values between the study group and the controls (correlation coefficient = 0.577; *p* = 0.012).

**Conclusions:**

After successful MH surgery, the FAZ area in both the SCP and DCP was smaller and the VD ratio of the DCP was lower, suggesting a possible DCP vulnerability to tractional stress. As the FAZ area of the DCP in closed-MH eyes became smaller than that in the controls, the fovea was less displaced toward the optic disc, possibly reflecting a lack of retinal redundancy caused by horizontal stretching accompanied by foveal displacement.

**Electronic supplementary material:**

The online version of this article (10.1186/s12886-017-0607-z) contains supplementary material, which is available to authorized users.

## Background

Our understanding of the pathogenesis, staging and recovery of the retinal architecture in patients with macular hole (MH) has been broadened with the development of optical coherence tomography (OCT). In particular, spectral-domain OCT (SD-OCT) has clearly identified, in postoperative eyes with MH, signs of anatomic recovery of retinal microstructure. These signs include the restoration of the photoreceptor inner segment and outer segment (IS/OS) junction and external limiting membrane (ELM) [1–3]. Thus, recent studies have focused on retinal-structural changes before and after surgery.

However, the tractional forces resulting in the development of MH might be strong enough to damage the retinal vasculature as well. Several studies have reported certain surgical complications, such as post-inner limiting membrane (ILM) peeling focal intraretinal hemorrhage or edema, that might affect retinal vasculature [4, 5]. Unfortunately, the lack of effective assessment tools has rendered clarification of the status of the pre- and post-MH-surgery retinal microvasculature difficult.

The recent advent of OCT angiography (OCTA) allows for noninvasive in vivo visualization of the retinal microvasculature. OCTA, significantly, can quantify the superficial capillary plexus (SCP) and deep capillary plexus (DCP) separately in retinal diseases. Recently, a few studies have utilized OCTA to document the features of foveal microvasculature in MH eyes [6–9] and to investigate related clinical factors such as central foveal thickness (CFT) [6–8]. However, the changes after successful MH surgery and the related clinical factors are still not completely understood.

Thus, in this study, we employed OCTA to describe the features of the foveal microvasculature and to determine the related clinical factors in eyes with surgically closed MH.

## Methods

This study was a retrospective case series. It was approved by the Institutional Review Board of Kangnam Sacred Heart Hospital, Hallym University, and was conducted in accordance with the tenets of the Declaration of Helsinki.

We retrospectively reviewed the medical records of patients who had undergone pars plana vitrectomy (PPV) for unilateral idiopathic full-thickness MH. We included patients who had maintained complete postoperative hole closure for at least 12 months as well as, for controls, healthy fellow eyes as determined by ophthalmoscopic and tomographic examinations. The exclusion criteria were as follows: any history or clinical evidence of chorioretinal diseases such as age-related macular degeneration, choroidal neovascularization, diabetic retinopathy, retinal vein or artery occlusion, uveitis in either eye, any optic nerve disease such as glaucoma in either eye, high myopia of spherical equivalent greater than −7.0 diopters or axial length (AXL) greater than 26 mm in either eye, abnormal vitreomacular interface in the fellow eye, a history of any intraocular surgery except uncomplicated cataract surgery in the fellow eye, and poor-quality OCTA images (of <50 signal strength index). Patients with systemic diseases that could affect the retinal microvasculature, such as uncontrolled diabetes mellitus or hypertension, also were excluded.

One of two vitreoretinal surgeons (SHB and HK) performed the standard 23-gauge PPV including indocyanine-green-assisted ILM peeling, a tamponade with perfluoropropane gas, and postoperative face-down positioning for 5 to 7 days. Simultaneous or subsequent cataract extraction was performed if needed.

**Fig. 1:**
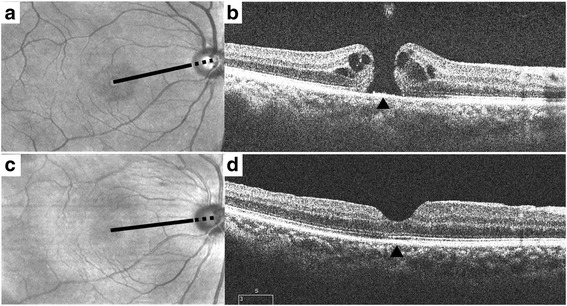
Fundus and optical coherence tomography (OCT) images of a 61-year-old woman with macular hole. **a** Preoperative fundus image; the distance from the margin of the optic disc to the center of macular hole was measured manually using the caliper of the OCT with a length of 4725 μm (black line). **b** Preoperative OCT image with full thickness MH; the arrowhead indicates the center of MH. **c** Postoperative fundus image 6 years after surgery; the distance between the temporal disc margin and the fovea was decreased to 4360 μm (black line). **d** Postoperative OCT image with closed MH; the arrowhead indicates the center of fovea

All of the patients underwent preoperative and postoperative comprehensive ophthalmologic examinations including slit-lamp biomicroscopy, dilated fundus examination and AXL measurements of both eyes. To measure the papillofoveal distance (PFD), we first drew a line from the center of the MH or fovea to the bifurcated central retinal artery within the disc; then, we manually measured the distance between the center of the MH or fovea and the point at which the line meets the temporal margin of the optic disc, using the built-in caliper of the OCT device (Fig. 1).

We obtained cross-sectional OCT images of both eyes using SD-OCT (Cirrus HD-OCT, Carl Zeiss Meditec, Jena, Germany) before and after surgery. A fovea-centered raster-scan protocol was applied. At the baseline, we measured the minimum and base diameter of the MH as well as its height [10]. After surgery, we measured the CFT through the foveal center on OCT images and evaluated the integrities of the outer retina, including the photoreceptor IS/OS junction and the ELM line, for 500 μm in both directions from that center.

After surgery, OCTA images of both eyes were acquired by swept-source OCT (SS-OCT; DRI OCT Triton, Topcon Corporation, Tokyo, Japan) with a central wavelength of 1050 nm, an A-scan rate of 100,000 scans per second, and an in-depth digital resolution of 2.6 μm. The instrument analyzed the OCT intensity information using a ratio method, namely OCTA Ratio Analysis, by which the full spectrum is maintained intact and the axial resolution is preserved. The OCTA images, as centered on the fovea, were obtained in 3 mm × 3 mm square scans at a 320 × 320 resolution.

We measured several OCTA parameters, including the area of the foveal avascular zone (FAZ) and the capillary vascular density (VD) ratio, in both the SCP and DCP. The built-in OCTA software was used to modify the automated segmentation in depth of retina manually to reduce the segmentation error and to outline the FAZ area in both the SCP and DCP. To calculate the VD ratio, the OCTA images were converted using the adjust threshold tool set to default by ImageJ software (National Institutes of Health, Bethesda, Maryland, USA), as previously reported [11]. The VD ratio was defined as the ratio of the total pixel area of 3 mm × 3 mm scanned area occupied by vessels in white pixels. OCTA-image features have been reported to vary widely: the mean FAZ area in the SCP, for example, has been shown to range from 0.25 to 0.474 mm^2^ [12–14]. Kita et al., meanwhile, found that the FAZ area in eyes with closed MH was significantly related to that in fellow eyes [8]. In the present study then, in order to offset the individual variations of OCTA-image features, we chose the FAZ-area and VD-ratio difference values between the study and fellow eyes as the OCTA parameters. The OCT and OCTA images were reviewed independently by two trained graders (JHC, HCY) masked to all clinical information. Any disagreement between them was resolved by consensus.

Statistical analyses were performed using SPSS software version 24.0 (IBM-SPSS, Chicago, Illinois, USA). The continuous variables were recorded as mean ± standard deviations and the categorical variables as percentages. Paired *t* tests were used to determine the OCTA-parameter differences between the study and fellow eyes. We analyzed the relationship between the OCTA parameters and several clinical factors including age at surgery, sex, AXL, MH stage, MH dimensions and PFD before and after surgery, postoperative CFT, and the integrities of the photoreceptor IS/OS junction and ELM. Spearman’s rank-correlation coefficient test was used to calculate the correlations between the OCTA parameters and the other continuous variables. We used the Mann-Whitney *U* or Kruskal Wallis test to compare the OCTA parameters according to the categorical variables. A *p* value less than .05 was considered statistically significant.

## Results

A total of 18 eyes of 18 patients with surgically closed idiopathic MH were included in this study. The mean age of the patients was 61.8 ± 9.9 years (range: 38–76); 7 (38.9%) were male and 11 (61.1%) were female. The preoperative MH stages were stage 2 in 5 eyes (27.8%), stage 3 in 12 eyes (66.7%), and stage 4 in 1 eye (5.5%). The mean time lag between surgery and OCTA-image acquisition was 38.6 ± 22.5 months (range: 12–76). After surgery, the mean PFD was significantly decreased, from 3776.7 ± 420.9 μm (range: 3021–4530) to 3625.3 ± 384.8 μm (range: 2905–4236) (*p* = 0.004), and the fovea was displaced nasally by 149.8 ± 243.9 μm (range: −123–978). The patients’ demographic and ophthalmic characteristics are summarized in Table 1.

**Table 1:** The demographic and ophthalmic characteristics of the participants

Characteristic	Participants
Number of eyes (patients), n	18 (18)
Age, y	61.8 ± 9.9 (38–76)
Sex, n (%)	
Men	7 (38.9)
Women	11 (61.1)
Axial Length, mm	23.5 ± 1.0 (21.9–24.9)
BCVA, logMAR	
preoperative	0.80 ± 0.29 (0.40–1.22)
postoperative at the time of OCTA acquisition	0.11 ± 0.12 (0.00–0.5)
Preoperative stage, n (%)	
Stage 2	5 (27.8)
Stage 3	12 (66.7)
Stage 4	1 (5.5)
Cataract surgery, n (%)	
Simultaneous	10 (55.6)
Sequential	5 (27.8)
Time lag between surgery and OCTA acquisition, mo	38.6 ± 22.5(12–76).
Preoperative SD-OCT measurements	
Minimum diameter of MH, μm	316.4 ± 146.4 (115–569)
Base diameter of MH, μm	632.7 ± 255.8 (188–1017)
Height of MH, μm	411.4 ± 49.8 (347–522)
Postoperative SD-OCT measurements	
Central foveal thickness, μm	185.3 ± 60.8 (30–298)
Foveal detachment, n (%)	0 (0)
Intact photoreceptor IS/OS junction, n (%)	12 (66.7)
Intact external limiting membrane, n (%)	12 (66.7)
Papillofoveal distance, μm	
Preoperative	3776.7 ± 420.9 (3021–4530)
Postoperative	3625.3 ± 384.8 (2905–4236)

Values are expressed as the mean ± standard deviation (range) unless otherwise indicated

*BCVA* best-corrected visual acuity, *IS/OS* inner segment and outer segment, *logMAR* logarithm of the minimum angle resolution, *MH* macular hole, *mo* month, *OCTA* optical coherence tomography angiography, *SD-OCT* spectral-domain optical coherence tomography

**Fig. 2:**
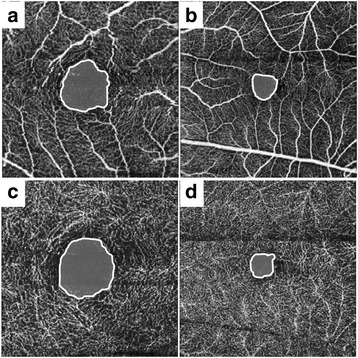
Foveal microvasculature features observed by optical coherence tomography angiography (OCTA) for closed macular hole (MH). **a** The OCTA image of the superficial capillary plexus (SCP) for a 40-year-old woman with the fellow eye; the foveal avascular zone (FAZ) is delineated by the white line with an area of 0.51 mm^2^. **b** The OCTA image of SCP for the closed-MH eye; the FAZ area is 0.24 mm^2^, which is smaller than that for the fellow eye. **c** The OCTA image of deep capillary plexus (DCP) for the fellow eye; the FAZ area is 0.56 mm^2^. **d** The OCTA image of DCP for the closed-MH eye; the FAZ area is 0.26 mm^2^, which is smaller than that for the fellow eye

On the postoperative OCTA images, the mean FAZ area in both the SCP and DCP for the closed-MH eyes (0.29 ± 0.11 mm^2^ and 0.39 ± 0.14 mm^2^) was significantly smaller than those for the fellow eyes (0.45 ± 0.14 mm^2^ and 0.62 ± 0.22 mm^2^) (*p* = 0.001 and <0.001, respectively). The mean VD ratio in the SCP for the closed-MH eyes (0.270 ± 0.349) was similar to that for the fellow eyes (0.321 ± 0.189) (*p* = 0.231); however, the mean VD ratio in the DCP for the closed-MH eyes (0.321 ± 0.189) was significantly lower than that for the fellow eyes (0.331 ± 0.119) (*p* = 0.025). The data are summarized in Table 2. A representative case is shown in Fig. 2.

**Table 2:** The foveal avascular zone area and vascular density in the superficial and deep capillary plexuses

	Superficial capillary plexus	Deep capillary plexus
Foveal avascular zone area, mm^2^		
Closed-MH eyes	0.29 ± 0.11 (0.11–0.44)	0.39 ± 0.14 (0.18–0.68)
Fellow eyes	0.45 ± 0.14 (0.18–0.74)	0.62 ± 0.22 (0.32–1.14)
*p* value, [95% CI]	0.001 [−0.23–−0.09]	<0.001 [−0.31–−0.13]
Vascular density, ratio		
Closed-MH eyes	0.270 ± 0.349 (0.223–0.325)	0.321 ± 0.189 (0.294–0.370)
Fellow eyes	0.283 ± 0.318 (0.232–0.349)	0.331 ± 0.119 (0.302–0.352)
*p* value, [95% CI]	0.231 [−0.03–0.01]	0.025 [−0.02–0.00]

Values are expressed as the mean ± standard deviation (range)

*CI* confidence interval, *MH* macular hole

**Fig. 3:**
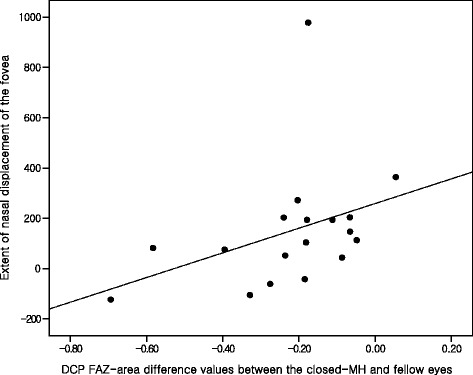
The relationship between the foveal displacement and the FAZ area in closed macular hole (MH). The scattergram shows that the extent of nasal displacement of fovea is significantly correlated with the DCP FAZ-area difference values between the closed-MH eyes and the fellow eyes (correlation coefficient = 0.577, *p* = 0.012). FAZ = foveal avascular zone, DCP=deep capillary plexus

Only the extent of nasal displacement of the fovea was correlated with the DCP FAZ-area difference values between the study and fellow eyes (correlation coefficient = 0.577; *p* = 0.012) (Fig. 3). The FAZ area of the study eyes and FAZ-area difference values in both the SCP and DCP did not show any significant relationship with the other clinical factors including age at surgery, sex, AXL, MH stage, minimum and base diameter as well as height of MH, PFD before and after surgery, extent of nasal displacement of the fovea, postoperative CFT, and the integrities of the IS/OS junction and ELM (*p* > 0.05) (see Additional file 1). No significant relationship between any of these clinical factors and the VD ratio of the study eyes or the VD-ratio difference values in either the SCP or DCP was revealed (*p* > 0.05) (see Additional file 2).

## Discussion

In this study, we investigated the OCTA-measured features of the retinal microvasculature in eyes with surgically closed MH as compared with their fellow eyes. Previously, several studies had evaluated the effect of tractional forces and vitreoretinal surgery on retinal microcirculation in several macular diseases including epiretinal membrane (ERM) and MH [15, 16]. For the eyes with ERM, reduced perifoveal capillary blood-flow velocity was reported based on fluorescein angiography with scanning laser ophthalmoscopy, which was gradually improved after ERM removal [15, 16]. OCTA images, meanwhile, have shown reduced choriocapillary circulation [17] and vascular sliding at the border of the cystoid cavities in eyes with MH [9]. These reports enabled us to infer the occurrence of disturbed macular microcirculation induced by vitreoretinal traction during the development of MH; note, however, that we did not evaluate the preoperative status of the retinal microvasculature.

After surgery, we observed, consistently with previous reports [6–8], a smaller FAZ area in the closed-MH eyes in both the SCP and DCP compared with the fellow eyes. Those earlier reports posited that a filling-in process with centripetal movement of the macular tissue might contribute to a smaller FAZ area in closed-MH eyes [6–8]. We concur. We would add that the smaller FAZ area might suggest that the damage to the macular microvasculature was not so severe as to irreversibly destroy the perifoveal capillary plexus, which condition would manifest as an enlarged FAZ.

Our results showed a significantly lower VD ratio in only the DCP, though we cannot explain the pathogenesis definitively. Coscas et al., having detected more severe damage to the DCP than to the SCP in eyes with retinal vein occlusion, hypothesized that the DCP is connected to the major veins through transverse venules, thus making it more vulnerable to increased intravascular pressure or reduced oxygen supply [18]. Although MH pathogenesis is not related to retinal vascular disorder, the DCP might yet be more vulnerable to tractional stress, or macular capillary network restoration could be more limited in the DCP even after successful MH closure.

In this study, we also evaluated the relationship between the OCTA parameters and several clinical factors for eyes with surgically closed MH. The fovea had been less shifted toward the optic disc, as the FAZ area of the closed MH in the DCP was smaller than in the fellow eyes. Nonetheless, none of the other clinical factors (i.e., age at surgery, sex, MH stage, MH dimensions such as the minimum and base diameter or height, PFD before and after surgery, postoperative CFT, and outer-retinal integrities such as the IS/OS junction and ELM) showed any significant relationship with the OCTA parameters.

A few previous studies have examined the relationship between OCTA parameters and several clinical factors in surgically closed MH such as CFT and ganglion cell-inner plexiform layer thickness [6–8]. However, studies investigating the related clinical factors are relatively lacking, and the results that have been obtained are inconsistent. Baba et al. [6] and Kita et al. [8] reported inverse correlations between the FAZ area and CFT, but Yun et al. [7] showed no significant correlation with mean retinal thickness. We also did not detect any correlation between the OCTA parameters and postoperative CFT. Yun et al., furthermore, reported a horizontally asymmetric VD ratio, suggesting, as an explanation, the influence of foveal displacement after ILM peeling, but they did not evaluate foveal displacement [7]. Several studies have reported that the PFD was shortened after vitrectomy with ILM peeling in eyes with MH and diabetic macular edema, showing that foveal displacement toward the optic disc leads to stretching and thinning of the retinal parenchyma in the temporal retina [19–22]. Two of those studies suggested, as a possible operative mechanism, the release of inherent forces in the retinal parenchyma or axonal shrinkage of the nerve fibers after ILM peeling [21, 22]. Yoshikawa et al. [22] proposed “less retinal elasticity” as an explanation for the fact that their observed PFD shortening did not extend to the outer retina. Considering these earlier results together with our own, we hypothesize that the foveal displacement toward the optic disc induced stretching of the temporal inner retina after ILM peeling, resulting in reduced additional centripetal movement of the inner retina into the fovea due to a lack of tissue redundancy. Additionally, the horizontal stretching to the temporal inner retina initiated by foveal displacement might damage the deeper-layer DCP more severely than the superficial-layer SCP, which might result, in turn, in delayed postoperative remodeling and restoration of the DCP rather than the SCP.

This study has several limitations, two of which are its small number of subject eyes and retrospective nature. Also, we did not evaluate the serial changes of the macular microvasculature over time before and after surgery; the follow-up period, moreover, was variable, ranging from 12 to 76 months. The duration of anatomical recovery after MH surgery might be minimal after a year [23], but we cannot rule out further changes to clinical factors and OCTA parameters. We should also note that we manually measured the FAZ area and several structural features such as the MH dimensions and PFD, which could have led to measurement bias. Steel D et al. have reported that the size of ILM peel area was significantly related with change in disc to foveal distance in patients with MH [24]. Thus, the area of ILM peel might be a relevant factor in changes of macular microvasculature, but we did not measure the ILM peel size in this study. Further studies with a larger sample size, a serial follow-up before and after surgery and measurement of ILM peel area are needed to support our results.

## Conclusions

Our results confirmed smaller FAZ areas in both the SCP and DCP after successful MH surgery, but the VD ratio in the DCP was lower than that for the fellow eyes, suggesting a possible vulnerability to tractional stress in the DCP. Additionally, as the FAZ areas in the DCP were smaller for the closed-MH eyes than for the fellow eyes, the fovea had been less displaced toward the optic disc, suggesting a lack of retinal redundancy caused by horizontal stretching with foveal displacement. Further studies are needed for support of our results and assumptions.

## Additional files

Additional file 1: Table S1. The relationship between the clinical factors and the foveal microvasculature parameters of surgically closed macular hole including the foveal avascular zone (FAZ) area and the FAZ-area difference values between the study and fellow eyes. (DOCX 15 kb)

Additional file 2: Table S2. The relationship between the clinical factors and the foveal microvasculature parameters of surgically closed macular hole including the vascular density (VD) ratio and the VD-ratio difference values between the study and fellow eyes. (DOCX 15 kb)

## Abbreviations

AXL: Axial length
CFT: Central foveal thickness
DCP: Deep capillary plexus
ELM: External limiting membrane
ERM: Epiretinal membrane
FAZ: Foveal avascular zone
ILM: Inner limiting membrane
IS/OS: Inner segment and outer segment
MH: Macular hole
OCT: Optical coherence tomography
OCTA: Optical coherence tomography angiography
PFD: Papillofoveal distance
PPV: Pars plana vitrectomy
SCP: Superficial capillary plexus
SD-OCT: Spectral-domain optical coherence tomography
SS-OCT: Swept-source optical coherence tomography
VD: Vascular density
