# Supplementary material for: Foveal microvasculature features of surgically closed macular hole using optical coherence tomography angiography
Source: BMC Ophthalmol. 2017 Nov 28;17:217. doi: 10.1186/s12886-017-0607-z (PMC5704531; doi:10.1186/s12886-017-0607-z)
Supplement: Supplementary file 1 — The relationship between the clinical factors and the foveal microvasculature parameters of surgically closed macular hole including the foveal avascular zone (FAZ) area and the FAZ-area difference values between the study and fellow eyes. (DOCX 15 kb) [file 12886_2017_607_MOESM1_ESM.docx]

Additional file 1: **Table S1** The relationship between the clinical factors and the foveal microvasculature parameters of surgically closed macular hole including the foveal avascular zone (FAZ) area and the FAZ-area difference values between the study and fellow eyes.

| Clinical factors | FAZ area  in the SCP | | FAZ area  in the DCP | | FAZ-area difference value in the SCP | | FAZ-area difference value in the DCP | |
| --- | --- | --- | --- | --- | --- | --- | --- | --- |
|  | Correlation coefficient | *p* value | Correlation coefficient | *p* value | Correlation coefficient | *p* value | Correlation coefficient | *p* value |
| Preoperative factors |  |  |  |  |  |  |  |  |
| Age at surgery | -0.211 | 0.401 | 0.103 | 0.683 | -0.113 | 0.656 | 0.366 | 0.135 |
| Sex |  | 0.425 |  | 0.791 |  | 0.536 |  | 0.151 |
| Axial length | -0.018 | 0.943 | 0.117 | 0.645 | 0.042 | 0.867 | 0.057 | 0.823 |
| MH stage |  | 0.443 |  | 0.566 |  | 0.384 |  | 0.075 |
| Minimum diameter of MH | 0.296 | 0.233 | 0.152 | 0.548 | -0.049 | 0.548 | -0.01 | 0.968 |
| Base diameter of MH | 0.17 | 0.499 | 0.169 | 0.502 | -0.13 | 0.502 | -0.135 | 0.593 |
| Height of MH | 0.232 | 0.353 | 0.169 | 0.502 | 0.196 | 0.502 | 0.363 | 0.139 |
| Preoperative PFD | -0.156 | 0.537 | -0.09 | 0.723 | 0.154 | 0.542 | 0.317 | 0.2 |
| Postoperative factors |  |  |  |  |  |  |  |  |
| Duration between surgery and OCTA acquisition | 0.256 | 0.306 | 0.297 | 0.231 | 0.268 | 0.282 | 0.104 | 0.68 |
| Postoperative PFD | 0.046 | 0.855 | -0.057 | 0.823 | 0.125 | 0.622 | -0.061 | 0.81 |
| Extent of nasal displacement of fovea | -0.384 | 0.115 | -0.185 | 0.463 | -0.006 | 0.98 | 0.577 | 0.012^*^ |
| Central foveal thickness | -0.467 | 0.05 | -0.189 | 0.453 | -0.445 | 0.064 | 0.129 | 0.610 |
| Integrity of the photoreceptor IS/OS junction |  | 0.291 |  | 0.083 |  | 0.892 |  | 0.75 |
| Integrity of the ELM |  | 0.291 |  | 0.083 |  | 0.892 |  | 0.752 |

*DCP* deep capillary plexus, *ELM* external limiting membrane, *FAZ* foveal avascular zone, *IS/OS* inner segment and outer segment, *MH* macular hole, *PFD* papillofoveal distance, *OCTA* optical coherence tomography angiography, *SCP* superficial capillary plexus

* *P* < 0.05
