# Supplementary material for: Foveal microvasculature features of surgically closed macular hole using optical coherence tomography angiography
Source: BMC Ophthalmol. 2017 Nov 28;17:217. doi: 10.1186/s12886-017-0607-z (PMC5704531; doi:10.1186/s12886-017-0607-z)
Supplement: Supplementary file 2 — The relationship between the clinical factors and the foveal microvasculature parameters of surgically closed macular hole including the vascular density (VD) ratio and the VD-ratio difference values between the study and fellow eyes. (DOCX 15 kb) [file 12886_2017_607_MOESM2_ESM.docx]

Additional file 2: Table S2. The relationship between the clinical factors and the foveal microvasculature parameters of surgically closed macular hole including the vascular density (VD) ratio and the VD-ratio difference values between the study and fellow eyes.

| Clinical factors | VD ratio  in the SCP | | VD ratio  in the DCP | | VD-ratio difference value in the SCP | | VD-ratio difference value in the DCP | |
| --- | --- | --- | --- | --- | --- | --- | --- | --- |
|  | Correlation coefficient | *p* value | Correlation coefficient | *p* value | Correlation coefficient | *p* value | Correlation coefficient | *p* value |
| Preoperative factors |  |  |  |  |  |  |  |  |
| Age at surgery | -0.35 | 0.155 | -0.313 | 0.205 | 0.012 | 0.961 | -0.054 | 0.832 |
| Sex |  | 0.246 |  | 0.085 |  | 0.659 |  | 0.596 |
| Axial length | -0.432 | 0.073 | -0.084 | 0.742 | 0.073 | 0.773 | -0.147 | 0.562 |
| MH stage |  | 1.0 |  | 0.336 |  | 0.289 |  | 0.075 |
| Minimum diameter of MH | -0.001 | 0.997 | 0.37 | 0.131 | 0.146 | 0.564 | 0.320 | 0.195 |
| Base diameter of MH | 0.061 | 0.81 | 0.271 | 0.276 | 0.075 | 0.766 | 0.299 | 0.227 |
| Height of MH | -0.229 | 0.360 | -0.372 | 0.129 | -0.274 | 0.272 | 0.016 | 0.950 |
| Preoperative PFD | 0.042 | 0.868 | -0.284 | 0.254 | 0.073 | 0.773 | -0.053 | 0.836 |
| Postoperative factors |  |  |  |  |  |  |  |  |
| Duration between surgery and OCTA acquisition | 0.344 | 0.162 | 0.395 | 0.104 | 0.184 | 0.465 | 0.358 | 0.145 |
| Postoperative PFD | 0.195 | 0.438 | 0.071 | 0.779 | 0.108 | 0.669 | -0.160 | 0.526 |
| Extent of nasal displacement of fovea | -0.13 | 0.607 | -0.345 | 0.161 | -0.202 | 0.421 | 0.149 | 0.556 |
| Central foveal thickness | -0.346 | 0.16 | -0.032 | 0.9 | -0.249 | 0.32 | 0.36 | 0.142 |
| Integrity of the photoreceptor IS/OS junction |  | 0.892 |  | 0.964 |  | 0.82 |  | 0.385 |
| Integrity of the ELM |  | 0.892 |  | 0.964 |  | 0.82 |  | 0.385 |

*DCP* deep capillary plexus, *ELM* external limiting membrane, *IS/OS* inner segment and outer segment, *MH* macular hole, *PFD* papillofoveal distance, *OCTA* optical coherence tomography angiography, *SCP* superficial capillary plexus, *VD* vascular density
